# Molecular Factors Involved in the Reproductive Morphophysiology of Female Domestic Cat (*Felis catus*)

**DOI:** 10.3390/ani13193153

**Published:** 2023-10-09

**Authors:** Luciano Cardoso Santos, Juneo Freitas Silva

**Affiliations:** Nucleo de Pesquisas em Reproducao e Endocrinologia, Centro de Microscopia Eletronica, Departamento de Ciencias Biologicas, Universidade Estadual de Santa Cruz, Campus Soane Nazare de Andrade, Ilheus 45662-900, Brazil; lucianouesc280@gmail.com

**Keywords:** uterus, ovary, placenta, immune factors, angiogenesis, domestic cat

## Abstract

**Simple Summary:**

This article presents the current knowledge regarding the modulation and expression profile of hormonal, immunological, redox, and growth mediators involved in the reproductive morphophysiology of the domestic cat.

**Abstract:**

The domestic cat (*Felis catus*) is considered an important model for the study of feline reproductive morphophysiology. However, although the morphological changes and clinical signs that occur during the estrous cycle and pregnancy are well known, little is known about the molecular mechanisms involved in the reproductive physiology of this animal species. Thus, this paper reviews the current knowledge about the modulation and expression profile of hormonal, immunological, redox, and growth mediators involved in the uterine, ovarian, and placental morphophysiology of domestic cats.

## 1. Introduction

The domestic cat (*Felis catus*) is considered an important model for studying the reproductive morphophysiology of felines [1]. Moreover, studies have shown the relevance of this research for assisted reproductive techniques (ARTs) in wild cats [2]. According to the International Union for Conservation of Nature (IUCN), at least half of the feline species are classified as being at some level of risk on the Red List (28.94% (11/38) vulnerable, 15.78% (6/38) near threatened, and 13.15% (5/38) endangered [3]). Furthermore, hunting, slaughter, and habitat destruction are among the main causes listed for the reduction of the population of these animals [3].

Among the feline species, the domestic cat is the most widely studied, along with the cheetah (Acinonyx jubatus) [1]. Female cats are known to exhibit both copulation-induced ovulation [4] and spontaneous ovulation, which can occur even in the absence of genital stimuli [5,6] and in at least one-third of queens [6]. These cycles can be ovulatory or not [7] but generally depend on reproductive seasonality since cats are a seasonal polyestrous species [8]. However, although this species shows greater cyclicity on long days, as observed during spring and summer, queens can cycle throughout the year and not have seasonal anestrus periods due to photoperiod modifications in temperate and near-tropical climates [9]. In addition, the increase in the cat populations in cities has become a public health problem. Therefore, the knowledge of their reproductive physiology serves as a basis for establishing new population control strategies.

In the domestic cat, the estrous cycle is divided into five phases: proestrus, estrus, interestrus, diestrus, and anestrus [8,10], the latter of which is the phase of reproductive quiescence [11]. The diagnosis of the phase can be made by vaginal smear, and not all these phases can be easily differentiated histologically in the female cat [12,13]. In particular, the proestrus and estrus are similar in terms of histology of the ovary and uterus, and they are differentiated according to the clinical and behavioral characteristics of the animal. Thus, for routine histological characterization, only the anestrus, proestrus/estrus, and diestrus are usually considered [14].

When the cat ovulates but fertilization does not occur, the cycle shifts to the diestrus phase (luteal phase), with an average duration of between 35–45 days [8]. When fertilization occurs, the subsequent pregnancy lasts, on average, 65 days from copulation [15]. Cats have a circular zonary placenta that forms a belt around the fetus. The fetal membranes at the maternal–fetal interface are endotheliochorial in structure, with moderate trophoblast invasion within the endometrium [16,17].

Although the morphological changes and clinical signs that occur during the estrous cycle and pregnancy are well known, little is known about the molecular mechanisms involved in the reproductive physiology of this animal species. Studies conducted in the last decade with domestic cats have shown that several growth, hormonal, immunological, and redox factors are expressed differently in the uterus and ovary throughout the reproductive cycle [14,18,19,20,21,22,23,24,25], as well as in the placenta throughout pregnancy [18,19,26,27]. Moreover, these studies show that changes in the expression of some of these factors are involved in the reproductive alterations observed in pyometra [14,28,29,30] and in feline immunodeficiency virus (FIV) infection [31,32,33,34]. Thus, this paper reviews the current knowledge about the modulation and expression profiles of hormonal, immunological, redox, and growth mediators in the uterus, ovary, and placenta of domestic cats throughout the estrous cycle and during pregnancy.

## 2. The Estrous Cycle of Domestic Cats

**Figure 1:**
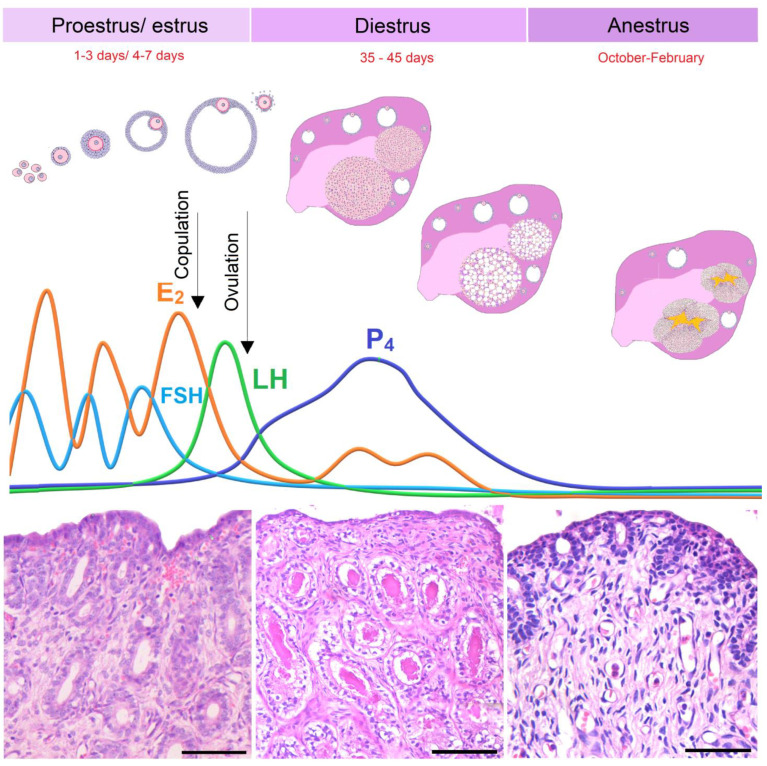
Hormonal profile in the ovulatory cycle of the domestic cat. In the domestic cat’s estrous cycle, E_2_ levels increase gradually, concomitantly with high FSH levels, and reach the highest values at the beginning of the estrus phase. In the proestrus/estrus phase, concomitant with the growth of ovarian follicles, the uterus has numerous growing glands and prominent vascularity (**lower left panel**). Usually, after copulation, there is an increase in the concentration of LH, leading to ovulation. In other cases, without ovulation, cats go through an interestrus phase and return to the proestrus phase. With ovulation, the LH surge leads to the formation of the corpus luteum, which initiates P_4_ production. In the diestrus phase, the uterus (**lower central panel**) presents endometrial glands in secretory activity. P_4_ levels fall progressively towards the end of the luteal phase. The anestrus phase is characterized by a low hormonal profile, which shows a phase of reproductive quiescence (**bottom right panel**). Legends: E_2_ = estradiol; P_4_ = progesterone. Staining: Hematoxylin and Eosin. Bar: 50 µm.

The estrous cycle in cats comprises the proestrus, estrus, interestrus, diestrus, and anestrus phases [8,10]. The characteristics of each phase are marked by behavioral changes in the queens, accompanied by hormonal and morphological changes in the genital tract [8]. In the proestrus, which lasts an average of one or three days [8], rapid follicular growth occurs under stimulation from the follicle-stimulating hormone (FSH) (Figure 1) [10,35], synthesis and secretion of estrogens by the ovary, and endometrial proliferation. This phase also includes the behavioral characteristics of rubbing the head and neck on objects and the attraction of the male, roaming but without receptivity to copulation [8,10,35].

The estrus lasts an average of four to seven days [8] and is the period in which the female is receptive to the male due to the highest serum levels of estradiol (E_2_) produced by the ovaries, the peak of which reaches ~60 pg/mL [36]. Queens often express strong vocalization, and some of them may urinate inappropriately. The hormonal changes are important for the vascularization and growth of the endometrium, especially the endometrial glands [35] (Figure 1). At the end of the estrus, ovulation does not commonly occur, which leads the queens to reduce their estrogen synthesis to basal levels, resulting in a period called interestrus that lasts 1 and 3 weeks without receptivity to the male [8,35].

After the preovulatory luteinizing hormone (LH) surge, if ovulation occurs, the cycle transitions to the diestrus (luteal phase). In this period, circulating progesterone (P_4_) levels increase, with a peak of ~24 ng/mL [36], due to the formation of the corpus luteum (CL) in the ovary [8,37,38]. The uterus exhibits the luminal epithelium with high columnar cells, sometimes with a pseudostratified or hyperplastic appearance, in addition to numerous glands that are branched to the base of the endometrium [39,40] (Figure 1). Mild to moderate dilatation of the uterine lumen also occurs, as well as glandular secretory activity with fluid accumulation inside the uterus [35,41]. If fertilization occurs, the cats become pregnant, and the luteal phase is maintained for around 65 days [10,35], with the P_4_ levels ranging from ~7.8 to 5.1 ng/mL between days 30 and 60 of pregnancy [42].

**Figure 2:**
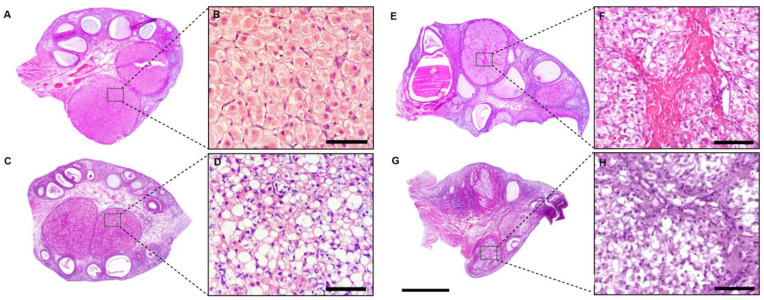
Main maturation stages of the corpus luteum of the domestic cat. (**A**,**B**) Ovary with mature corpus luteum showing voluminous and eosinophilic luteal cells (highlight (**B**)); (**C**,**D**) ovary showing a mature corpus luteum with vacuolation of luteal cells (highlight (**D**)); (**E**,**F**) ovary with corpus luteum in initial regression showing smaller, retracted luteal cells with central fibrous tissue formation (highlight (**F**)); (**G**,**H**) ovary of an animal in anestrus, with evident corpus *albicans* (highlight (**H**)) (Staining: Hematoxylin and Eosin. Bar: (**A**–**D**) = 2 mm; (**E**–**H**) = 50 µm).

In the absence of pregnancy, the luteal phase is maintained for approximately 35–45 days [10,43]. At the end of this period in non-pregnant cats in diestrus, the CL undergoes a period of slow luteal degeneration (luteal regression) while, in cats at the end of pregnancy, there is a rapid and intense luteal degeneration (luteolysis), similar to what occurs in bitches. [44,45], leaving a remnant scar in the ovarian tissue called the corpus *albicans* (Figure 2H), which will be reabsorbed [44]. Despite the difference in the CL functionality time between non-pregnant animals in the diestrus and pregnant animals, in both cases, this gland exhibits similar morphological changes throughout the luteal phase [44]. An early stage of luteal formation can be observed, with smaller and more basophilic luteal cells, followed by the development and maintenance of a mature CL, evidenced by larger, polyhedral, and more eosinophilic luteal cells (Figure 2A,E). Subsequently, these cells undergo an early and late regression, with cytoplasmic vacuolation (Figure 2B,F), connective tissue formation (Figure 2C,G), and leukocyte infiltration, followed by the formation of the corpus *albicans* (Figure 2D,H), which exhibits a large deposition of connective tissue and retracted and vacuolized lutein cells [44] (Figure 2H).

With the regression of the CL, in regions where seasonality is well defined, the queens move into an anestrus, which is an inactive phase of the reproductive cycle and which usually occurs between the months of October and February [8], or they start a new cycle if it is still breeding season. Anestrus is characterized by reduced levels of E_2_ (6–12 pg/mL [36]) and P_4_ (<1 ng/mL [36]) and higher levels of melatonin (9226 ± 1052 pg/mL) and prolactin (164 ± 5 ng/mL) [46] since this phase is associated with short periods of luminosity [47]. The ovary does not contain mature follicles and corpus luteum, and the endometrium is lined with a thin luminal epithelium, with two or three layers of inactive glands located near the apex of the endometrium, as well as a higher proportion of stromal cells [40] (Figure 1). At this stage, therefore, the uterine and ovarian morphology reflects the inactivity of the cycle.

## 3. Placentation in Cats

**Figure 3:**
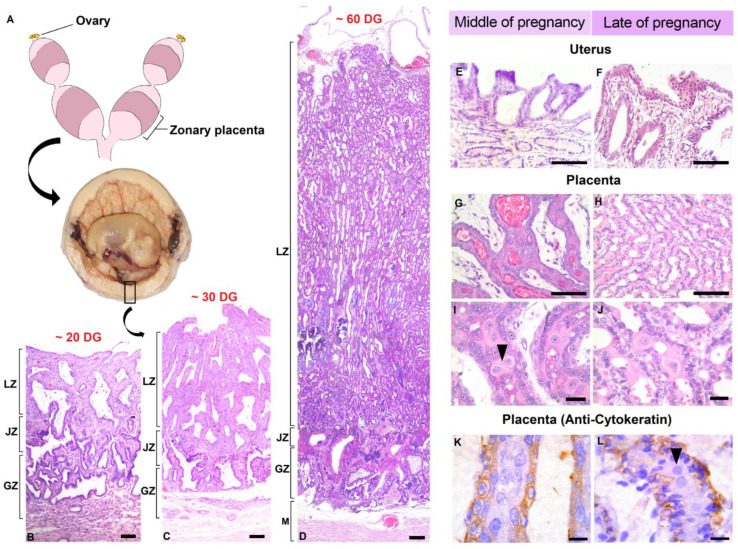
Endotheliochorial circular zonary placenta of the domestic cat. (**A**) Macroscopic characterization: circular region of the placenta. (**B**–**D**) Histology of the placenta at approximately 20 days (**B**), 30 days (**C**), and 60 days of gestation (**D**). (**E**,**F**) Endometrial histology in mid- (**E**) and late pregnancy (**F**). (**G**–**L**) Lamellar organization of the placenta in mid-pregnancy (**G**,**I**,**K**) and late pregnancy (**H**,**J**,**L**) shows organization of trophoblast and decidual giant cells (arrowheads). (**K**,**L**) Immunostaining of basic cytokeratin in cytotrophoblast and syncytiotrophoblast. Absence of staining in decidual giant cells (arrowheads). (Streptavidin-biotin-peroxidase, 1:400 dilution). (Staining: Hematoxylin and Eosin. Bar: (**B**–**D**) = 100 µm; (**E**,**F**) = 50 µm; (**G**–**L**) = 20 µm. Legends: DG = day of gestation; LZ = lamellar zone; JZ = junctional zone; GZ = glandular zone; M = myometrium).

In the female cat, implantation occurs around the 12.5th day post-copulation (dpc), followed by the beginning of the formation of the definitive zones of the placenta (14th and 15th dpcs), when the chorionic processes begin to invade the endometrium [48]. This invasion occurs through the endometrial crypts, and the process preserves the maternal structures that, together with the proliferation of the trophoblast, will give rise to the definitive placental lamellae until the 20th dpc [49] (Figure 3B).

The female domestic cat has a type of placentation referred to as endotheliochorial. This means the syncytiotrophoblast, the innermost and most invasive trophoblast layer, is in closer contact with the maternal endothelium. In this type of placentation, there is no clear separation between the maternal and fetal parts as occurs in the epitheliochorial placenta (e.g., equines and porcine), in which there is less decidual reaction when compared with endotheliochorial placentation [50,51,52,53]. In the endotheliochorial placenta and, more markedly, in the hemochorial (e.g., rodents), there is a greater trophoblastic invasion, leading to a moderate [54] and high decidual reaction, respectively. This makes tissue separation difficult and causes rupture of some of the maternal blood vessels, resulting in some injuries and bleeding when the placental tissue detaches during parturition [51].

Based on the pre-established classifications [48,50,51], the placenta of the domestic cat has characteristics that are common among most carnivores. In relation to the macroscopic shape of the placenta, the domestic cat has a belt around the chorionic sac, which is why it is called a zonary annular or circular placenta [49,50,51] (Figure 3A). Regarding the form of maternal–fetal interdigitation, the cat has a complex and branched lamellar-type structure [48,51,55]. The lamellae extend parallel from the base of the endometrium, where they are formed, to its surface on which large blood vessels and fetal tissues are distributed. These lamellae are uterine projections that grow towards the embryo as thin folds with maternal components. The placenta with a lamellar structure is formed by the glandular, junctional, and lamellar zones, which have distinct histological characteristics [56] (Figure 3B–D).

The glandular zone is formed by the surface of the endometrium and uterine glands. Then, the junctional zone functions as a link between the endometrium and the placenta [57]. It is in this layer that cytotrophoblast cells invade the endometrium and destroy epithelial cells but retain maternal capillaries and some stromal cells [57]. The stromal cells, which are surrounded by a non-transformed extracellular matrix, can be individualized or grouped in plates. In this layer, they are called decidual cells [54,57] since they come from fibroblasts of the uterine mucosa [56]. The junctional zone, in this sense, leads to the formation of definitive placental lamellae due to the association between decidual cells (Figure 3L; arrowhead) and maternal capillaries with proliferating trophoblasts [57].

The lamellar zone follows the junctional zone. Structurally, the lamellae are lined with trophoblasts, although maternal capillaries and cells of deciduous origin are located in the center [56] (Figure 3B–D). In this region, these cells are known as giant cells; however, they are morphologically and functionally similar to the decidual cells in the junctional zone [57]. Externally to the lamellar zone, chorion cells cover its entire extension, and in the middle of this chorionic layer, fetal blood vessels associate closely with trophoblasts. This entire structure involving the lamellae, chorion cells, and fetal vessels tends to increase in complexity throughout pregnancy, either by increasing the number of cells or by the high branching of the lamellae and vascularization [48,49,51].

The last classification for the feline placenta concerns the arrangement of maternal–fetal blood vessels. The domestic cat, which has endotheliochorial placentation, exhibits a simple countercurrent arrangement of the vessels [51]; that is, the blood flow is perpendicular.

## 4. Factors Associated with Uterine, Ovary, and Placental Morphophysiology of the Domestic Cat

Several hormonal, immunological, redox, and growth mediators are fundamental in the morphophysiology of the female genital system and participate in various processes of the reproductive cycle and pregnancy [58,59,60,61]. Thus, changes in the expression of these mediators have been observed in several pathological conditions in both women and animals, such as recurrent abortion [62,63], preeclampsia [64,65], premature birth [66,67], and pyometra [14,28,29,30], which can not only result in subfertility or infertility but also cause the death of the individual.

### 4.1. Hormonal Factors

#### 4.1.1. Pituitary Gonadotropins

It is well established that the domestic cat has an episodic secretion of LH and undergoes gonadal inhibitory influence (negative feedback) [68], which is similarly found in other species of spontaneous ovulation. In addition, the concentration of LH increases after copulation [4,69,70]. However, its secretion varies according to the period of estrus in which copulation occurs, with larger secretions reported at the beginning of the estrus [70,71], as well as with the number of copulations [4,69,71]. In addition to LH, FSH is also crucial for gonadal function in the domestic cat [72,73], both of which act on the ovary through its LHR and FSHR receptors, respectively. The expression of these receptors and the functions of LH/FSH were well characterized in the ovary of the domestic cat [74].

##### Ovary

In ovarian follicles in the early antral period, the FSHR expression is restricted to the granulosa cells, while LHR is expressed in theca cells but also granulosa cells of large follicles (>800 µm in diameter) and CLs [74,75]. When comparing prepubertal and adult cats, no differences are observed in the ovarian gene expression of *FSHR* and *LHR* or the LHR protein. However, a significantly lower expression of the FSHR protein was observed in prepubertal cats compared to adult luteal-phase cats [75].

Regarding the functions of these gonadotropins in the ovary, studies have shown that the administration of human FSH (huFSH) and human menopausal gonadotropin (hMG), followed by human chorionic gonadotropin (hCG) increased the number of follicles from 1 to 3 mm and induced ovulation, thus evidencing its role in the folliculogenesis of the cat [72]. Although GnRH and/or agonists are commonly used to suppress the reproductive cycle in queens and bitches [76], the administration of a single intramuscular dose of gonadorelin induces ovulation in queens when administered on days 2–4 after the onset of estrus [77]. In addition, the administration of FSH in female cats improves oocyte quality and the development of parthenogenic embryos [73,78]. A study also demonstrated that in the pre-implantation period in domestic cats, the luteal LHR concentration correlates positively with the CL mass and P_4_ concentration, suggesting an association between these factors in the maintenance of embryonic viability [79].

#### 4.1.2. Sex Hormones

In addition to the gonadotropins produced in the pituitary gland, hormones produced in the gonads are crucial for the reproductive morphophysiology of the female [80]. These hormones are called sex steroids and are mainly estrogens and P_4_, which also act on the organs of the female genital tract through their ER and PR receptors, respectively. However, recent studies in humans and other animal species have also demonstrated the importance of androgens, through signaling with their receptor (AR), in uterine morphophysiology [81,82], thus also suggesting its participation in the reproductive physiology of cats [25,27]. In the female cat, receptor expression for sex steroids has been well-documented in the uterus [25,83,84], in the ovary [84,85], and in the placenta [27].

##### Uterus and Placenta

In the uterus, the presence of estrogen receptor alpha (ERα) was characterized in cats in different phases of the estrous cycle, demonstrating that this receptor is expressed in all cell types but that, especially in the estrus and diestrus, its labeling is intense in the glandular epithelium [84]. This study showed that in the anestrus—the reproductive quiescence phase—labeling is weak in the lining epithelium and myometrium and moderate in the stroma and glands [84]. In contrast, in a recent study, a lower ERα expression was observed in all uterine compartments in the diestrus phase, while protein and gene expression were upregulated in the proestrus/estrus and anestrus [25].

As for PR, the studies of Binder et al. [83] and de Jesus Nascimento et al. [25] showed that its expression is lower in the uterus of diestrus cats when compared to cats in the proestrus/estrus and anestrus. In addition to PR, progesterone receptor membrane components 1 and 2 (PGRMC-1 and PGRMC-2) were evaluated in follicular- and luteal-phase cats [83]. This study showed that, in the endometrial epithelium, the expression of PGRMC-1 was lower in diestrus cats. However, PGRMC-2 expression was higher in the endometrial epithelium and lower in the endometrial stroma but did not differ between cats in the diestrus or follicular phase.

As for the androgen receptor (AR), its immunolabeling in the uterus of cats was mainly in the luminal and glandular epithelium and in the myometrium, regardless of the phase of the estrous cycle, with no significant difference between the phases in both immunolabeling and gene expression [25].

In the maternal–fetal interface, although the roles of sex steroids are well known, studies in domestic species, such as cats, are rare. The expression of PR and ERα was observed in the endometrium and myometrium of cats during pregnancy [86]. However, until recently, it was unknown whether pregnancy itself could influence the uterine expression of these receptors. In this regard, the study by de Jesus Nascimento et al. [27] showed that ERα is upregulated in the endometrium and myometrium of domestic cats in mid-pregnancy when compared to the endometrium of cats in late pregnancy or in the non-gestational diestrus. In addition, in this study, the immunolabeling area of AR and PR did not differ in the uterus of pregnant and non-pregnant cats. However, the *PR* gene expression was higher in mid-pregnancy in relation to late pregnancy and the non-gestational diestrus, while no variations were observed in *AR* gene expression [27]. Interestingly, increased cytoplasmic immunolabeling of AR and reduced nuclear labeling in the luminal and glandular epithelium was observed in pregnant cats compared to non-pregnant cats, suggesting that pregnancy in cats alters the intracellular localization of AR since the receptor was translocated from the nucleus to the cytoplasm [27].

In the placenta, while ERα and PR were not observed in the trophoblast, the decidual giant cells showed intense expression at the end of pregnancy. In contrast, AR expression was observed in all placental cell types, with higher immunolabeling in mid-pregnancy compared to late pregnancy [27]. Interestingly, the decidual cells also more intensely express the genes encoding the steroidogenic acute regulatory protein (*StAR*) and 3β-hydroxysteroid dehydrogenase/isomerase (*3βHSD*) enzymes in mid-to-late gestation [87], and domestic cat placental homogenates are able to produce P_4_, showing that the placenta acts as an additional source of P_4_ during the final third of gestation [87,88]. The study of Tsutsui et al. [88] showed that, in fact, the placenta of queens locally produces P_4_, but the levels are not essential for the maintenance of pregnancy in late gestation.

##### Ovary

The ERα expression was also characterized in the ovary of cats in different phases of the estrous cycle. In the estrus, moderate to intense expression was demonstrated in the epithelial lining, while the follicular and interstitial cells showed weak expression [84]. Weak ovarian ERα expression was also observed in cats in the anestrus and diestrus [84]. In addition to the gene expression of sex steroid receptors, the study of Kehoe et al. [89] evaluated the expression of several genes encoding steroidogenic enzymes and membrane transporters in early preantral follicles (primordial, primary, and secondary) and reported a high expression of these mediators in these phases.

The study of Amelkina et al. [85] also characterized the expression of genes encoding several receptors, such as *ESR1/ERα*, *ESR2/ERß*, *PGR/PR*, *AR*, *PGRMC-1*, and *PGRMC-2*, in the CL of pregnant and non-pregnant cats. Reportedly, some of these receptors, such as *PR*, *ERα*, and *PGRMC-1*, are upregulated at the onset of luteogenesis in pregnant cats, while others, such as *PR*, *ERα*, *AR*, and *PGRMC-2* have greater expression during luteal regression in non-pregnant cats. Together, these data demonstrate the involvement of these factors in the production/regulation of sex steroids in the ovary of domestic cats and show important differences in their modulation depending on the luteal phase. In addition, these studies shed light on the differences in the regulation of luteal function between domestic cats and other feline species [90].

#### 4.1.3. Anti-Müllerian Hormone (AMH)

Anti-Müllerian hormone (AMH) is highly known for its role in inhibiting the Müllerian ducts during embryonic development. In adult females, their roles are related to follicular development, and AMH levels increase with follicle growth. Therefore, this has been considered an important marker of ovarian function [91,92]. In the female domestic cat, there are variations in the AMH levels between and within individual cats, as well as between the cycle phases within single queens [93]; however, levels of this hormone are much higher in younger cats [93,94,95]. Castration in female cats reduces the circulating concentration of AMH [96,97,98], confirming that the ovary is, until now, the main known source of production of this hormone in females. Furthermore, when cats are treated with deslorelin for estrus induction, the AMH levels rapidly drop and return after implant removal [99]. This shows that the queen still has ovaries but is at rest [99]. AMH levels are also important for the diagnosis of ovarian remnant syndrome (ORS) [93], although studies show little relationship between the AMH concentrations and the pathology of ovarian cysts in queens [98].

##### Ovary

In the ovary of the cyclic and pregnant domestic cat, the immunolocalization of AMH and its type 2 receptor (AMHRII) was evaluated by Gültiken et al. [100]. In that study, the percentage of marked area for AMH was higher in cats at 41–46 days of gestation—the period when the serum levels of AMH were also high [100]. As for AMHRII, its expression did not differ during pregnancy in cats; however, all pregnant groups had a higher expression when compared to cats in estrus [100].

#### 4.1.4. Relaxin (RLN)

Relaxin (RLN) is a peptide belonging to the insulin superfamily, and its main functions are related to the maintenance of pregnancy [101]. In the pregnant queen, RLN levels are detected from the 20th day of gestation, with maximum levels around the 30th day [102]. Furthermore, an evaluation of RLN production showed that placental homogenates have higher production when compared to the uterus and CL [102]. Thus, commercial kits for detecting RLN can be used for pregnancy diagnosis [103,104,105].

##### Uterus and Placenta

In domestic cat placenta, *RLN* mRNA was detected in the trophoblast at 35 days of gestation but not in other placental or non-placental tissues [106]. The study of Braun et al. [107] also evaluated the gene expression of *RLN* and its receptor *RXRP1* in the placenta and other reproductive tissues of the queen throughout pregnancy. In fact, these authors also confirmed the placenta as the main site of *RLN* expression. Its receptor, on the other hand, presented low numbers of copies in the placenta throughout the gestation; however, in the uterus, its expression was high [107].

#### 4.1.5. Kisspeptin/Kiss1r System

Kisspeptin is known for controlling hypothalamic GnRH release [108]. In domestic cats, its expression was demonstrated in at least four populations of cell bodies in coronal sections: amygdaloid complex, anterior periventricular nucleus, the tubular component of the periventricular nucleus, and the infundibular nucleus, thus also suggesting central physiological functions in this species [109,110]. This expression was also demonstrated in the genital tract of female cats in organs such as the uterus [14,111], ovary [23,111], and placenta [26], suggesting the participation of this peptide in the local physiological regulation of these organs.

##### Uterus and Placenta

In the uterus, the expression of kisspeptin and its receptor has been demonstrated in two studies [14,111]. In the study of Tanyapanyachon et al. [111], Kiss1 and Kiss1r have been described in the luminal and glandular epithelia, in addition to the endometrial stroma (except Kiss1r), myometrium, and perimetrium. However, no differences were observed between the stages of the estrous cycle [111]. The study of Santos et al. [14], however, showed that Kiss1 and Kiss1r have higher endometrial immunolabeling in the diestrus and proestrus phases, respectively. Furthermore, this study showed a positive correlation between the expression of genes *KISS1* and *KISS1R* in the uterus of cats and reported that the pyometra, a major uterine inflammatory disease in female cats, increases endometrial protein expression of Kiss1 and Kiss1r [14]. These findings suggest that the uterine expression of Kiss1/Kiss1r in female cats is modulated by the estrous cycle and in the pyometra condition.

In pregnant cats, the uterine expression of Kiss1 and Kiss1r increases in late pregnancy compared to mid-pregnancy, as was also observed in the placental gene expression of *KISS1R* [26]. In the placenta, Kiss1 is mainly expressed in trophoblasts and decidual giant cells in mid-pregnancy, but its immunolocalization is restricted to the decidual giant cells in the placenta at full-term [26]. As for Kiss1r, its localization in the placenta mid-pregnancy is mainly in the syncytiotrophoblast, while in the full-term placenta, it is also restricted to decidual giant cells [26].

##### Ovary

Tanyapanyachon et al. [111] and Santos et al. [23] also characterized the ovarian expression of kisspeptin and its receptor in domestic cats. These studies showed that both Kiss1 and Kiss1r are expressed in all ovarian compartments. However, unlike Tanyapanyachon et al. [111], Santos et al. [23] demonstrated a smaller Kiss1 labeling area in the granulosa cells in preovulatory follicles and little Kiss1r expression in most stages of follicular development. Regarding gene expression, Tanyapanyachon et al. [111] showed that *KISS1* is reduced in the ovary of diestrus cats, even when lutein cells have high protein expression. Santos et al. [23], however, evaluated the gene expression in CLs and found that pregnancy increases the luteal expression of *KISS1* when compared to the CL of non-pregnant cats, while luteal regression reduces the gene expression of *KISS1* and *KISS1R*. In immunolabeling, mature and regressing CLs express Kiss1 and Kiss1r, and pregnancy also increases the immunolabeling of Kiss1 and Kiss1r in the lutein cells of mature CLs when compared to non-pregnant cats in the diestrus [23]. This finding suggests the participation of this system in the luteal activity of cats during pregnancy.

In a recent study, Loncová et al. [112] cultured granulosa cells from follicles of prepubertal cats in the follicular phase with Kisspeptin-10 (Kp-10) and observed its effects on viability, cell proliferation, apoptosis, and steroid hormone release. These authors showed that incubation with Kp-10, despite not affecting cell viability, increased proliferation, apoptosis, and the production of P_4_ and E_2_, thus characterizing, for the first time, the action of kisspeptin in the domestic cat ovary.

### 4.2. Growth Factors and Proteases

Growth factors and proteases are molecules that participate in important cellular processes such as division, differentiation, cell proliferation, and protein synthesis and have drawn attention for their use in regenerative medicine [113]. These factors can be of various types, such as epidermal growth factors (EGFs) [114], insulin-like growth factors (IGFs) [115], fibroblastic growth factors (FGFs) [116], transforming growth factors (TGFs) [117], vascular endothelial growth factors (VEGF), [118] and matrix metalloproteinases [119], and were documented in the reproductive morphophysiology of the domestic cat (Table 1).

#### 4.2.1. Uterus and Placenta

Growth factors are still poorly studied in the uterus and placenta of domestic cats. It was initially shown that the insulin-like growth factor-binding protein 1 (IGFBP-1) has high expression at the sites of embryonic implantation [120], suggesting its participation in a trophoblastic invasion of the endometrium. Subsequently, another study demonstrated the immunolocalization of transforming growth factor Æ (TGF-Æ), epidermal growth factor (EGF), and its receptor (EGFR) in the endometrium and placenta of cats [121], where the endometrial expression of TGF-Æ increased after treatment with P_4_. TGF-Æ and EGF are important for angiogenesis and endometrial remodeling [122,123]. During pregnancy in the cat, both were expressed mainly in deep endometrial glands, while EGFR was expressed in a smaller amount between days 10 and 18 post-copulation. In the placenta, these factors were expressed in syncytiotrophoblast and decidual giant cells, while no expression was observed in the trophoblasts in late pregnancy [121].

**Table 1:** Gene and/or protein regulation of growth mediators and proteases in the uterus and placenta of the domestic cat.

Tissue	Estrous Cycle Stage/Gestational Stage	Main Findings	Ref.
Endometrium and placenta	Pregnant or non-pregnant cats (All stages)	IGFBP-1 (IHC) in implantation sites from the 16th gestational day.	[120]
Endometrium and placenta	Pregnant cats (All stages)	*↑* TGF- Æ (IHC) after stimulation with E_2_ + P_4._	[121]
Endometrium and placenta	Pregnant or non-pregnant cats (All stages)	*↑ Igf2* (RT-qPCR) in the pregnant uterus; *↑ Mmp2* and *Egf* (RT-qPCR) in the post-implantation uterus.	[19]
Endometrium	Non-gestation, pre-implantation, implantation, early and mid-pregnancy	*↑ Igf1* (RT-qPCR) in pre-implantation, implantation, and early pregnancy; *↓ Igf2* (RT-qPCR) in pre-implantation; *↓ Igfr1* (RT-qPCR) in mid-pregnancy; *↑ Igfbp1* and *Igfbp3* (RT-qPCR) in early and mid-pregnancy; *↓ Igfbp4* (RT-qPCR) in pre-implantation; *↑ Igfbp5* (RT-qPCR) in mid-pregnancy.	[20]
Endometrium	Non-gestation, early, mid-, and late pregnancy	*↑ Vegf* (RT-qPCR) in the interplacental region in late pregnancy.	[18]
Endometrium	Early pregnancy	*Paf1β*, *Paf1γ*, *Paf:ah* e *Pafr* (RT-qPCR) expression in early pregnancy.	[124]
Endometrium	Proestrus/estrus, diestrus, anestros	*↑* VEGF and Flk-1 (IHC) in proestrus/estrus or diestrus compared to anestros; *↑ Vegf* and *Plgf* (RT-qPCR) in the diestrus stage.	[14]

Legends/Signals: ↑ = Increase/High; ↓ = Reduce/Low; RT-qPCR = quantitative reverse transcription PCR; IHC = immunohistochemistry.

Subsequently, two other studies described the expression of growth factors in the endometrium of non-pregnant cats and at different times of pregnancy. Agaoglu et al. [19] showed that EGF, transforming growth factor β (TGF-β), insulin-like growth factor 2 (IGF-2), and IGF-2 receptor (IGF2R) are expressed in the uterus and placenta of domestic cats and that the *IGF2* had higher gene expression in the pregnant endometrium. Moreover, genes of the IGF family, such as *IGF1*, *IGF2*, *IGFBP1*, *IGFBP3*, and *IGFBP4*, had high uterine expression in early and mid-pregnancy, while *IGFR2* and *IGFBP2* did not differ between the gestational stages [20]. Studies in humans have shown that IGFs, especially IGF-2, act on pre-implantation embryonic development and promote proliferation and trophoblastic invasion [125].

In addition to EGF and IGF, the uterus and placenta of female cats express VEGF and hypoxia-inducible factor 1-α (HIF-1α), a factor that acts in angiogenesis by stimulating VEGF activity [18]. According to this study, the *HIF-1α* gene is reduced in the uterine samples from placental sites in mid-pregnancy, and it is highly expressed in the uterine samples from oocyte-positive cats (7 days after ovulation induction with GnRH analogs). Furthermore, the results showed that the *HIF-2α* gene was reduced in the uterine samples from embryo-positive female cats (7 days after copulation) but increased in the uterine regions linked to placental sites [18]. In addition, *VEGF* showed higher uterine gene expression in the interplacental region of cats in late pregnancy [18]. In female cats, studies have also demonstrated the uterine expression of platelet-activating factor (PFA) and its receptor (PFA-R) in pre-implantation, implantation, and throughout pregnancy [124], all of which are functionally important factors in early pregnancy [126,127].

VEGFs receive special attention because they are the main mediators involved in angiogenesis [128,129,130]. However, to carry out their function, they signal through their receptors VEGFR1 (Flt-1) and VEGFR2 (Flk-1/KDR) and act with other mediators, such as placental growth factor (PLGF), FGF, and angiopoietins (ANGs) [131]. All of them are involved in the reproductive morphophysiology of the female since angiogenesis is crucial not only in preparing the uterus for pregnancy but also for placentation [128,129,132,133].

It has been recently demonstrated that there is immunolocation and gene expression of VEGF and its receptor 2 (VEGFR2/Flk-1/KDR) in the uterus of pregnant and non-pregnant domestic cats, in addition to the gene expression of *PLGF* [14]. Both VEGF and Flk-1 are expressed mainly in the luminal and glandular epithelia of cats, with greater expression in the proestrus/estrus and diestrus phases when compared to the anestrus. In terms of genes, both *VEGF* and *PLGF* have greater uterine expression in the diestrus phase [14], suggesting that these factors are important for preparing the uterus for pregnancy. These results corroborate those of a previous study, in which it was demonstrated that both VEGF/*VEGF* and Flk-1 have higher endometrial expression in cats in mid-pregnancy than in late pregnancy and in the non-pregnant uterus in the diestrus phase [26]. In the placenta, VEGF/*VEGF* and Flk-1 also have higher expression in mid-pregnancy than in late pregnancy, and they are expressed in the syncytiotrophoblast and cytotrophoblast and mainly in decidual giant cells. In contrast, the *PLGF* showed increased placental gene expression in late pregnancy, while pregnancy reduced the uterine gene expression of *PLGF* compared to non-pregnant cats in the diestrus phase [26].

Together, these studies demonstrate that growth factors in the uterus and placenta of domestic cats are expressed differently throughout the estrous cycle, both temporally and spatially, with greater expression in the luteal phase and during pregnancy, thus suggesting the importance of these mediators in the placentation process. In addition to these assessments, these factors were also evaluated in the uterus of cats with pyometra [14]. In these animals, the endometrium considerably increases the labeling of the Flk-1 receptor, while there is a significant reduction in the gene expression of *VEGF* and *PLGF* [14], showing that the alteration of these factors is important in the pathogenesis of feline pyometra.

#### 4.2.2. Ovary

Angiogenesis is also a crucial process for the proper functioning of the ovary since an adequate formation of the vascular network allows the availability of oxygen, nutrients, hormones, and growth factors for the developing follicles and the CL [134]. In the follicle, granulosa cells are the main source of angiogenic factors [134]. Moreover, the development of primordial (avascular) follicles depends, in principle, on the proximity of blood vessels in the stroma for their nutrition [130]. Although preantral follicles express LH and FSH receptors [89], the initial growth of the follicles depends mainly on the growth factors produced locally in the ovary [135,136].

In this regard, studies have shown, in vitro, that the preantral follicles of domestic cats cultured with EGF or IGF-1 developed and exhibited oocyte maturation [137,138,139,140]. Moreover, the cultivation of primordial follicles from cats with FGF-2 promoted the activation and growth of these follicles and increased the diameter of the oocyte [141]. In addition, the in vitro use of EGF or insulin-transferrin-selenite (ITS) prevented the loss of cell viability of preantral follicles in female cats [142]. Similarly, an in vitro supplementation with EGF and/or IGF-1 increased the meiotic maturation of oocytes obtained from the ovaries of cats in the follicular phase [143], while high concentrations of EGF impaired the in vitro maturation of follicles [144]. Interestingly, the effects of IGF-1 on in vitro follicular maturation do not occur in follicles in advanced stages of development as they occur in small follicles [145], thus reinforcing the role of these factors mainly in preantral follicles.

In addition to these factors, VEGF and its receptor Flk-1 are expressed in the ovarian follicles of cats at all stages of development, while in preantral follicles, VEGF showed an intense expression in the oocyte and granulosa cells [23]. The cell signaling pathways with which these factors are associated have also been described. EGF helps maintain the in vitro viability of primordial follicles in cats by stimulating the signaling pathways of mitogen-activated protein kinase (MAPK) and phosphoinositide 3-kinase (PI3K), which, in turn, promote the proliferation of follicular cells [146]. Notably, a greater number of genes exhibit differential expression (DGEs) in the primordial-to-primary follicle transition in relation to the primary-to-secondary transition, especially for the genes associated with the PI3K-Akt and TGF-β pathways, erythroblastoma (ErbB) pathway, HIF-1 pathway, and matrix metalloproteinases [147].

In turn, studies on the expression and function of growth factors in antral and preovulatory follicles in domestic cats are scarcer. In relation to VEGF and its receptor Flk-1, intense labeling has been described in tertiary and preovulatory follicles [23]. This suggests that these mediators are also involved in follicular selection and the ovulatory process, as more intense changes involving ovarian angiogenesis are concentrated during ovulation [148].

The CL starts to form when the follicle is ruptured, and the resulting structure requires angiogenesis and the reorganization of blood vessels for the development of its endocrine function [130,149]. In this regard, the luteal phase is associated with an intense process of ovarian angiogenesis due to the high proliferation of endothelial cells [150]. Göritz et al. (1996) demonstrated that the concentration of EGF is higher in the CL when compared to other regions of the ovary of cats. Moreover, protein expression was observed not only in the CL but also in interstitial and internal theca cells. This same study also showed that EGFR has higher expression in the interstitial cells and the internal theca of tertiary follicles [151]. According to our observations, the mature CLs of cats in the early and late stages of regression express VEGF and Flk-1, while an upregulation occurs with the aging of the CL, especially for the VEGF [23]. This study also demonstrated that CLs of pregnant cats have a higher cytoplasmic marking of Flk-1 when compared to CLs of non-pregnant diestrus cats. In contrast, the gene expression of *PLGF* did not differ between the CL samples of cyclic and pregnant cats in relation to the corpus *albicans* [23].

Follicular development, ovulation, and luteogenesis also depend on the reorganization of the extracellular matrix. In this regard, matrix metalloproteinases (MMPs) are fundamental in these processes and in the modulation of immune responses [152]. Fujihara et al. [21] have shown that some MMPs, such as *MMP1* and *MMP3*, have higher gene expressions in the initial antral follicles of cats, while *MMP2* and *MMP9* have greater expressions in the early antral and antral follicles. *MMP7*, on the other hand, shows a minor variation in its expression between the types of follicles, while *MMP13* has greater expression in the primary follicles. These data suggest that MMPs are important proteolytic factors in domestic cat folliculogenesis and express differently according to the follicular stage.

Together, these studies demonstrate that the expression of growth factors and proteases in the ovary of female cats occurs mainly in two situations. First, this expression occurs in the maintenance and growth of preantral follicles, which depend on locally produced factors. Second, it occurs in the formation, maintenance, and regression stages of the CL, all of which require intense tissue remodeling.

### 4.3. Immune Factors

The immune system plays an essential role in reproduction, and several processes are dependent on its cellular and biochemical components. In the domestic female cat, as in other mammals, this system acts from the uterine and ovarian morphological changes that occur during the estrous cycle and during pregnancy [14,26]. Therefore, any dysregulation in the expression of these mediators is closely related to important pathological conditions in domestic cats, such as uterine hyperplasias and pyometra [14], in addition to infections caused by FIV [33,34,153].

#### 4.3.1. Uterus and Placenta

Studies have shown that interferon-gamma (INFγ), macrophage migration inhibitory factor (MIF), tumor necrosis factor-alpha (TNFα), and interleukin 10 (IL-10) have higher expressions in the uterus of cats in the proestrus/estrus and diestrus than in the anestrus, while *INFγ* and *MIF* have a higher gene expression in the proestrus/estrus and diestrus phases, respectively [14] (Table 2). Jursza et al. [28] also demonstrated that TNFa has higher endometrial expression in cats in the estrus, while Siemieniuch et al. [154] described lower gene and protein expressions of TNF in the interestrus. In this study, TNFα receptors (TNFR1 and TNFR2) were expressed in the uterus of the female cat, with *TNFR1* showing greater gene expression in the diestrus and *TNFR2* showing no differences between the phases of the cycle [154].

TNFα is a pro-inflammatory cytokine produced mainly by macrophages and acts in several pathophysiological processes in the body. In cats, it is involved in the production of prostaglandins (PGs) during the estrous cycle [28,154]. The in vitro administration of TNFα in the endometrial tissue of estrus and diestrus cats increased the secretion of prostaglandin 2α (PGF_2α_) and prostaglandin E2 (PGE_2_), respectively [154], which are similar effects to those found by Jursza et al. [28] and Jursza-Piotrowska et al. [155].

PGs play a key role in female reproduction, from ovulation, the transport of oocytes, fertilization, the passage of the embryo through the uterine tube, and maintenance of pregnancy [156]. The domestic cat has a high endometrial gene expression of various types of PGs, such as prostaglandin F synthase (*PGFS*), prostaglandin E synthase (*PGES*), and prostaglandin-endoperoxide synthase 2 (*PTGS2*) in the diestrus phase, especially in the mid-diestrus [157,158]. Possibly, these PGs are involved in the local hormonal response in the endometrium in queens during estrus and the luteal phase [158]. In fact, the expression of PGs is modulated throughout the estrous cycle and stimulated after the administration of estrogen and/or progesterone [158,159]. However, in this species, PGs are not considered important luteolytic factors since hysterectomy does not affect ovarian function [160], and diestrus cats exhibit lower expression of the protein PTGS2 in the endometrium and uterine tube [83].

Thus, despite the few studies on the expression of immune mediators throughout the estrous cycle in domestic cats, the factors described so far are expressed differently throughout the estrous cycle and are associated with physiological changes in the uterus of cats. Furthermore, studies have shown that alterations in the expression of these mediators are involved in some reproductive pathological conditions in cats, such as pyometra. Prostaglandin levels, especially PGF2α, are elevated in cats with pyometra [155,161,162]. Recently, it has also been demonstrated that the gene expression of *TNFα* is enlarged in the uterus of cats with pyometra [14], similar to that observed by Jursza et al. [28]. These data possibly explain the high gene levels of *PGFS* and *PTGS2* observed in cats with pyometra, in addition to the high concentration of PGF2α [155,157].

In addition, TNFα and lipopolysaccharide (LPS) increase the in vitro expression of Toll-like receptors (TLRs) 2 and 4 in the endometrial explants of cats in the estrus or those treated with P_4_, thus suggesting the participation of TLRs in pyometra of cats [163], as also demonstrated by Jursza-Piotrowska and Siemieniuch [29]. Activation of these receptors is important for triggering the innate immune response, which is an essential cellular defense mechanism in bacterial infections such as pyometra. Interestingly, a reduction in interleukin 6 (*IL-6*) gene expression and macrophage migration inhibitory factor (*MIF*) in the uterus of cats with pyometra, as well as an increase in the endometrial immunolabeling of IL-10, was observed [14], suggesting the activation of anti-inflammatory defenses.

In addition to participation in the estrous cycle and pyometra, immunological factors act on uterine receptivity during the embryonic implantation of carnivores and other mammals [164]. In domestic cats, the profile and function of different immune mediators were described in the uterus throughout pregnancy, mainly early, mid-, and late pregnancy. In the early stages of pregnancy, proteins such as desmoglein and e-cadherin, which help to reduce cell-to-cell adhesion and enable uterine blastocyst invasion, are redistributed in the uterine luminal epithelium of female cats in the pre-implantation period [165]. Furthermore, the uterine gene expression of platelet-activating factors (PAF), such as PAF 1 beta (*PAF1β*), 1 gamma (*PAF1γ*), PAF acetylhydrolase (*PAF:AH*), and PAF receptor (*PAFR*), was observed mainly in the implantation period of the cat [124]. The PAF pathway is well-documented in pathophysiological processes, including its role in embryonic implantation [166]. In addition, genes encoding the transcription factor forkhead box protein 3 (*FOXP3*) and cytotoxic T-lymphocyte antigen 4 (*CTLA4*), which are regulatory T-lymphocyte markers, show high expressions in the endometrium of cats in early pregnancy [167]. In contrast, a greater endometrial expression of INFγ, MIF, and TNFα was found in cats in mid-pregnancy than in late pregnancy, as well as a greater gene expression of *TNFα* and *IL-10* in the uterus of pregnant cats than in non-pregnant diestrus cats [26]. All these studies demonstrate that immune mediators express differently in the endometrium of cats throughout pregnancy, and pregnancy itself stimulates the endometrial expression of inflammatory mediators.

**Table 2:** Gene and/or protein regulation of immunological mediators in the uterus and placenta of the domestic cat.

Tissue	Estrous Cycle Stage/Gestational Stage	Main Findings	Ref.
Endometrium	Anestrus, proestrus/estrus, diestrus, and pyometra	*↑* INFγ, MIF, and TNFα (IHC) in proestrus/estrus and diestrus in relation to anestrus; *↑ Infγ* (RT-qPCR) in proestrus/estrus stage; *↑ Mif* (RT-qPCR) in the diestrus stage.	[14]
Endometrium	Estrus and diestrus	*↑* TNFα (IHC) in estrus; *↓* TNFα (IHC) in diestrus; *↑* TNFα (IHC) in deep glands after P_4_ administration.	[28]
Endometrium	Estrus, diestrus, and interestrous	*↓ Tnf* (RT-qPCR) and TNFα (WB) in the interestrous; *↑ Tnfr1* (RT-qPCR) in diestrus; *↑* PGF_2α_ [ ] at estrus and PGE_2_ [ ] at diestrus after 12 h incubation with TNF.	[154]
Endometrium	Mid- and late-luteal stage	*↑ Pgfs*, *Pges*, and *Ptgs2* (RT-qPCR) in mid-luteal stage.	[158]
Uterus and uterine tubes	Cats with or without CL in the ovary	*↓* PTGS2 (IHC) in cats with CL versus cats without CL.	[83]
Endometrium	Mid-luteal stage	*↑* PGF_2α_ [ ] after stimulation with AA, E_2_, and E_2_/P_4_ in epithelial cells. *↑* PGE_2_ [ ] after stimulation with AA and E_2_ in epithelial cells; *↑* PGF_2α_ and PGE_2_ [ ] after stimulation with AA in stromal cells.	[159]
Endometrium	Early and Late Pregnancy	*↑ Foxp3* and *Ctla4* (RT-qPCR) in early pregnancy.	[167]
Endometrial culture	Anestrus, estrus, mid- and late diestrus	*↑ Pges* and *Ptgs2* (RT-qPCR) after incubation with TNFα or LPS; *↑ Pgfs* (RT-qPCR) after incubation with TNFα or LPS; *↑* PGE_2_ [ ] after incubation with LPS or TNFα; *↑* PGF_2α_ [ ] after incubation with LPS or TNFα.	[155]
Uterus and placenta	Diestrus, mid- and late pregnancy	*↑* INFy, TNFα, and MIF (IHC) in mid-pregnancy uterus; *↑ Tnf* and *Il-10* (qPCR) in the pregnant uterus; *↑* INFγ, TNFα, and IL-10 (IHC) in the placenta in mid-pregnancy; *↑ Infγ*, *Il-6*, and *Il-10* (qPCR) in the placenta in mid-pregnancy.	[26]
Uterus	Anestrus, estrus, and late diestrus	*↑ Pge_2_* (qPCR) in late diestrus in relation to anestrus.	[29]
Endometrial culture	Estrus, mid- and late diestrus, anestros	*↑ Tlr2* (qPCR) after 2 h and 12 h of incubation with TNFα; *↑ Tlr4* (qPCR) after 2 h of incubation with TNFα; *↑ Tlr2* (qPCR) in estrus and late diestrus after incubation with TNFα; *↑ Tlr4* (qPCR) in estrus after incubation with TNFα.	[163]

Legends/Signals: ↑ = Increase/High; ↓ = Reduce/Low; RT-qPCR = quantitative reverse transcription PCR; WB = Western blotting; IHC = immunohistochemistry; [ ] = concentration.

In the cat placenta, the presence of immune mediators has been well characterized in some diseases, such as in FIV infection, while its expression in physiological conditions has still been scarcely studied. In FIV-positive cats, the expression of *IL-5*, *IL-1Β*, *IL-10*, and *TGF-β* was lower in early pregnancy [33,34,168], and the expression of *IL-6* and *IL-12P35* was greater in early pregnancy [33]. In the full-term placenta, however, a reduction was observed in *IL-4*, *IL-12P35*, *IL-12p40*, and *IL-1Β* [33,34] and an increase in *Il-6* [33]. In addition to these changes, Chumbley et al. [153] demonstrated that the placental gene expression of *FOXP3* and *RORΓ* was reduced in early pregnancy in FIV-infected cats, suggesting an imbalance in T-lymphocyte and T-lymphocyte *helper* 17 (Th17) populations at this gestational stage.

Regarding gestational success, some studies have also associated the dysregulation of placental immune mediators in FIV-positive cats with problems in fetal viability. Weaver et al. [31] showed that the genes *INFγ* and *IL-1β*, which are important products of the Th1 lymphocytes, are reduced in the placentas of FIV-positive cats with fetal resorption, as also described by Coats et al. [32]. In addition, a non-viable fetus in FIV-positive cats was also associated with reduced placental levels of *TGF-β*, *IL-10*, *IL-2*, *IL-6*, *IL-17a*, and *IL-1β* in early pregnancy. Similarly, Scott et al. [33] described the reduction of several cytokines in early pregnancy (*IL-4*, *IL-5*, *IL-6*, *IL-1β*, *IL-12P35*, *IL-12P40*, and *CXCR4*) or late pregnancy (*IL4*, *IL-12P35*, and *IL-12P40*). Chumbley et al. [153] also demonstrated the reduced placental expression of *IL-10*, *IL-2*, *IL-17a*, *IL-6*, and *TGF-β* in FIV-positive cats with non-viable fetuses in early pregnancy.

Taken together, these studies demonstrate that FIV infection in cats reduces the expression of placental immune mediators, especially in early pregnancy. This probably compromises fetal viability since physiologically high placental expression of immune mediators, such as *IL-6*, *IL-10*, and *IL-12P35*, occurs in cats in early pregnancy [33]. In contrast, *IL-6*, *IL-10*, *TNFα*, and *INFγ* maintain a high placental expression in mid-pregnancy relative to the full-term placenta [26], which is a gestational period with a high expression of *IL-1β* and *SDF-1A* [33].

In relation to PGs, a study showed that they have a high expression in the placenta of cats in early pregnancy and gradually reduce until delivery [169]. However, this same study noted that *PTGS2* has a high expression in the full-term placenta, which was also observed for the plasma concentration of 13,14-dihydro-15-keto-prostaglandin F2α (PGFM), an inactive metabolite of PGF_2α_ [169].

#### 4.3.2. Ovary

Although some studies have demonstrated that immune factors play several roles in ovarian function [170,171], these mediators are poorly studied in the ovary of the domestic cat. Stroma-derived factor 1 (SDF-1/CXCL12) and C-X-C chemokine receptor type 4 (CXCR4/CD184) have been observed in the cumulus–oocyte-complex (COC) in the domestic cat ovary [22], wherein SDF-1/CXCR4 binding is well known for the immune responses that result in ovulation [172]. To verify whether SDF-1/CXCR4 is also important in the ovulation of the domestic cat, cultures of COCs were made with SDF-1. According to the results of this study, SDF-1 increases the expression of some genes participating in the ovulatory cascade, such as *HAS2* and *TNFAIP6*, suggesting that the SDF1-CXCR4 pathway may play a direct role within the COC [22].

Recently, it was shown that the cytokines INFγ and MIF are expressed in ovarian follicles in domestic cats at all stages of development, and their location is mainly in the oocyte, granulosa cells, and theca [23]. Notably, both cytokines showed intense expression in the oocyte until the secondary follicle stage, suggesting that these factors may participate in important processes in the initial development of follicles [23].

Santos et al. [23] also evaluated the presence of INFγ and MIF in luteogenesis and luteal regression in pregnant and non-pregnant cats. Regarding gene expression, no differences were observed between the levels of *INFγ* in the corpus luteum (CL) samples from pregnant, non-pregnant, and corpus *albicans* (CA), while *MIF* showed lower gene expression in the CL of pregnant cats and in the CA. However, through immunohistochemical analysis, MIF in pregnant cats showed a higher expression in younger CLs [23].

In another study conducted by Amelkina et al. [173], the participation of the immune mediators was also evaluated in the luteogenesis and luteolysis of the domestic cat. This study demonstrated that some factors associated with the intrinsic apoptosis pathway, such as the genes *BAX*, *BCL2*, and *CASP3*, do not exhibit differences between the stages of luteogenesis and luteal regression in pregnant and non-pregnant cats. However, genes such as *FAS* and *TNFRSF1b*, which encode factors of the extrinsic pathway of apoptosis, have an increased expression at the onset of luteal regression. Thus, these genes possibly act on luteal regression in pregnant and non-pregnant cats.

In addition to these factors, the expression of PGs was evaluated in the luteogenesis and luteolysis of domestic cats, given their great importance for luteal function in this species [90]. The study of Zschockelt et al. [24] showed that the gene expression of *PTGS2/COX2*, *PTGES*, *PGFS*, and the receptor of *PTGER2* did not differ in the stages of luteogenesis and luteal regression in pregnant and non-pregnant cats. It has also been observed that genes *PTGER4* and *PTGFR* did not differ in the stages of luteogenesis in non-pregnant cats, but the CLs in late regression showed a higher expression of *PTGER4* than the CLs in development/maintenance. *PTGFR* expression was also higher in the luteal stages from maintenance to regression when compared to the period of CL formation [24].

### 4.4. Redox Mediators

Oxidative stress (OS) is a condition of cellular stress caused by an imbalance between the production of reactive oxygen species (ROS) and antioxidant defenses [174]. In domestic cats, however, studies involving factors linked to OS are scarce.

#### 4.4.1. Uterus and Placenta

Studies on female dogs submitted to an ovariohysterectomy showed an increase in the concentration or enzymatic activity of some OS markers in plasma after castration, such as thiobarbituric acid reactive substances (TBARS), malondialdehyde (MDA), and the enzymes glutathione peroxidase (GPx), superoxide dismutase (SOD), and catalase (CAT) [175,176,177]. In contrast, the activity of antioxidant enzymes and increased OS did not differ in the plasma of cats after castration [178].

In a recently published study, however, de Jesus Nascimento et al. [25] demonstrated that the antioxidant enzymes SOD1 and CAT have a high expression in the endometrium of cats in the diestrus phase when compared to the proestrus/estrus and anestrus. Moreover, the immunolabeling of CAT was weak or null in the proestrus/estrus and anestrus. These findings suggest the participation of these enzymes in the preparation of the uterus for pregnancy. In addition, a positive correlation between the genes *SOD1* and progesterone (*PR*) receptor was observed in the uterus of these animals, suggesting that SOD1 expression may be modulated by P_4_. In the subsequent study, de Jesus Nascimento et al. [30] showed a trend of increasing the *SOD1* gene in uterine samples of cats with pyometra, which showed an increase in P_4_ concentration. This study also demonstrated that pyometra reduces the endometrial expression of CAT, positively regulates the gene and protein expression of GPX1, and increases the expression of 8-hydroxyl-2’-deoxyguanosine (8-OhdG), which is an indicator of oxidative DNA damage [179]. These results indicate the occurrence of uterine oxidative stress in cats with the condition of pyometra.

In the maternal–fetal interface of cats, the modulation of antioxidant factors was also recently demonstrated by de Jesus Nascimento et al. [27]. In this study, a uterine upregulation of the *SOD1* gene was observed in mid-pregnancy, but no variation was observed in protein expression compared to non-pregnant cats. In relation to CAT, the protein expression in uterine samples of pregnant cats is lower when compared to non-pregnant cats in the diestrus; however, its expression is higher in the endometrium in late pregnancy in relation to mid-pregnancy [27]. GPX1, in turn, had lower endometrial immunolabeling in late pregnancy, while gene expression was higher in the pregnant uterus compared to the non-pregnant uterus [27]. Thus, this study demonstrated that antioxidant enzymes in the maternal–fetal interface of cats are modulated differently according to the gestational stage, and pregnancy stimulates the uterine gene expression of *SOD1* and *GPX1*, which are possibly important for the maintenance of pregnancy.

#### 4.4.2. Ovary

Braun et al. [180] quantified the expression of several antioxidant factors in the luteogenesis of pregnant and non-pregnant cats. Despite their expression, the genes encoding peroxidasin (*PXDN*), peroxiredoxin 6 (*PRDX*6), thioredoxin (*TXN*), thioredoxin reductase 2 (*TXNRD2*), and glutaredoxin 3 (*GLRX3*) did not differ regarding the stage of luteal development in pregnant and non-pregnant cats. Other genes, such as superoxide dismutase 1 (*SOD1*), glutathione peroxidase 4 (*GPX4*), and glutathione S-transferase P (*GSTP*), also showed no difference in the luteogenesis of pregnant cats. However, the expression of *GSPT* is lower in the CL under development/maintenance, while *SOD1* and *GPX4* have lower expression in the CL in the late regression of non-pregnant cats [180]. In addition to these results, this study showed that the genes encoding the enzymes catalase (*CAT*) and superoxide dismutase 2 (*SOD2*) have a high expression in the CLs of late stages of pregnant cats (*SOD2* and *CAT*) and non-pregnant cats (*SOD2*), thus suggesting its participation in luteal maintenance in this species [180]. Another study also demonstrated that supplementation of the ovarian transport medium with the enzyme SOD can reduce cell apoptosis, increase the survival of the COC, and improve embryo production in domestic cats [181]. These results confirm the importance of antioxidant enzymes in follicular development and oocyte viability in the species.

## 5. Conclusions

The domestic cat is considered an important experimental model for studying the reproductive morphophysiology of other felines, especially considering that many of these species are vulnerable or threatened with extinction. However, many specific characteristics of the reproduction of domestic cats, such as the molecular mechanisms that regulate the reproductive pathophysiology of the queens, need to be clarified. In recent decades, several studies involving the cat’s genital tract have been developed, mainly evaluating the uterus and ovary. The results of these studies show that several factors are expressed and participate in the functions of these organs, such as growth factors and proteinases, immune factors, and redox mediators. The modulation of most of these factors is dependent on the phases of the estrous cycle, and they can be positively or negatively regulated by conditions such as pyometra or infections caused by FIV. Similarly, the results of more recent studies have shown the expression and regulation of these factors during pregnancy, particularly their expression in critical periods of pregnancy, such as implantation and delivery. However, most of these studies use descriptive approaches for the expression of these factors, resulting in a shortage of functional studies that evaluate their participation in the reproductive morphophysiology of domestic cats.

## Data Availability

Not applicable.
